# Milk Powder Fortified with Folic Acid and Colostrum Basic Protein Promotes Linear Growth and Improves Bone Microarchitecture in Juvenile Mice Without Adverse Metabolic Effects

**DOI:** 10.3390/nu17243819

**Published:** 2025-12-05

**Authors:** Hongjuan Liu, Yixin Zhang, Yuanjue Wu, Wenbo Wan, Jiawen Liang, Hui Xiong, Liping Hao, Ting Xiong

**Affiliations:** 1 Department of Nutrition and Food Hygiene, School of Public Health, Guangzhou Medical University, Xinzao, Panyu District, Guangzhou 511436, China; d202381883@hust.edu.cn (H.L.);; 2 Department of Nutrition and Food Hygiene, Hubei Key Laboratory of Food Nutrition and Safety and the Ministry of Education (MOE) Key Laboratory of Environment and Health, School of Public Health, Tongji Medical College, Huazhong University of Science and Technology, 13 Hangkong Rd, Wuhan 430030, China; 3 Mead Johnson Nutrition and Health Innovation Institute, No. 28, Zhujiang Road East, Zhujiang New Town, Guangzhou 510623, China; ruby.wan@mjngc.com (W.W.);

**Keywords:** milk powder, folic acid, colostrum basic protein, linear growth, bone microarchitecture, juvenile mice

## Abstract

**Background:** The juvenile-pubertal period is a critical window for linear growth and bone mass accumulation. This study investigated the joint effects of folic acid (FA) and colostrum basic protein (CBP)-fortified milk powder on growth, bone health, and metabolic safety in juvenile mice. **Methods:** Three-week-old C57BL/6J mice (n = 120) were acclimatized for 1 week and then randomly assigned to three isocaloric diet groups for an 8-week intervention starting at 4 weeks of age: Control (AIN-93M), Milk (AIN-93M + FA/CBP-fortified milk powder), and Positive Control (AIN-93G). Body length and weight were measured twice weekly. Bone microarchitecture was assessed by micro-computed tomography, and bone remodeling was evaluated through histology and serum biomarkers. The GH–IGF-1 axis and related metabolic parameters were also assessed. **Results:** FA–CBP–fortified milk powder significantly accelerated linear growth at intervention week 2, with body length higher in the Milk group than in the Control group (*p* < 0.01). After 8 weeks, the Milk group showed improved trabecular bone mass and microarchitecture compared with Control, especially in males (*p* < 0.01). Bone remodeling was transiently elevated at intervention week 4, as indicated by higher serum osteocalcin and CTX-I, and by increased osteoclast and cartilage matrix formation versus Control (*p* < 0.05). The GH–IGF-1 axis was also temporarily activated at week 4, with elevated serum GH and IGF-1/IGFBP-3 ratio compared with Control (*p* < 0.05). These skeletal benefits occurred without excess weight gain or adverse metabolic effects compared with Control (all *p* > 0.05). **Conclusions:** FA-CBP-fortified milk significantly enhanced linear growth during puberty and improved bone mass and microstructure in early adulthood. These skeletal benefits are consistent with the transient activation of the GH–IGF-1 axis. Importantly, no adverse metabolic effects were detected from early intervention through adulthood, supporting its potential application in growth-promoting nutritional strategies.

## 1. Introduction

Childhood and adolescence are critical windows for linear growth and bone development, yet global progress in these areas remains uneven. Stunting, defined as a height-for-age z-score more than two standard deviations below the WHO reference median, still affects approximately 25% of children worldwide and is linked to elevated mortality, infection risk, and neurodevelopmental deficits [1,2,3]. Although China reports a lower stunting prevalence [4], recent national surveillance indicates a slowing trend in height gain among adolescents [5]. This emerging pattern highlights persistent challenges in achieving optimal linear growth and underscores the need for effective nutritional interventions.

Bone health in early life is equally crucial. Pediatric bone fragility disorders, characterized by mineralization defects and trabecular structural abnormalities [6], contribute to an estimated 33 million annual fractures globally [7]. More than 90% of peak bone mass is achieved before adulthood, and poor bone accrual increases the risk of osteoporosis later in life [8]. This disease affects over 200 million individuals and causes around 9 million fragility fractures annually [9]. Addressing childhood stunting and bone health in tandem is therefore a pressing public health priority.

Nutrition is one of the most critical modifiable factors influencing both linear growth and bone health [10]. Dairy products provide a bioavailable nutrient matrix and an ideal platform for nutrient fortification research. Colostrum basic protein (CBP) has been consistently shown to stimulate osteoblast proliferation, enhance calcium utilization, and improve bone density [11,12]. In contrast, the role of folic acid (FA) in bone biology remains underexplored. Beyond its established functions in hematopoiesis and neural tube defect prevention [13,14], emerging evidence suggests that FA may modulate bone metabolism through regulation of homocysteine, collagen maturation, and vascular function [15,16]. Epidemiological studies further indicate that adequate FA intake supports bone mineral density and reduces osteoporosis risk [17,18,19,20]. Recent mechanistic research proposes that FA may act via TGR5–AMPK signaling and NF-κB/ERK pathway regulation [21]. However, experimental evidence evaluating FA supplementation and bone outcomes remains limited.

Based on these complementary properties, we hypothesized that combined FA–CBP fortification in milk powder would enhance linear growth and skeletal health during critical developmental periods. To test this hypothesis, we evaluated linear growth, skeletal outcomes, and metabolic responses in a juvenile mouse model, aiming to provide experimental evidence supporting targeted nutritional strategies for improving skeletal development in children and adolescents.

## 2. Materials and Methods

### 2.1. Animals and Experimental Design

All animal procedures were approved by the Institutional Animal Care and Use Committee at Tongji Medical College, Huazhong University of Science and Technology, Wuhan, China (IACUC approval No. 4724) and in accordance with the ARRIVE guidelines. A total of 120 specific pathogen-free (SPF) C57BL/6J mice (60 males and 60 females, 3 weeks old) were obtained from Wuhan Shubeili Science and Technology Co., Ltd., Wuhan, China. Mice were housed under SPF conditions (Facility License No. SYXK[E]2024-0046) at 20–26 °C, 40–70% relative humidity, and a 12 h light/dark cycle, with ad libitum access to deionized water and experimental diets. After a 7-day acclimatization period, mice were randomly assigned to three groups (n = 40 per group, 20 males and 20 females): Control, Milk, and Positive Control.

### 2.2. Diets and Supplementation

All diets were formulated and manufactured by Jiangsu Synergetic Medical Bioengineering Co., Ltd., Nanjing, China (composition shown in Tables S1 and S2). The Control group received the standard AIN-93M diet, which is formulated for maintenance and contains lower protein and nutrient levels suitable for mice. The Positive Control group was provided with AIN-93G, a diet specifically formulated for juvenile growth, containing higher protein content and key micronutrients to support rapid skeletal development [22]. AIN-93G served as a reference for maximal growth potential, enabling evaluation of whether FA+CBP-fortified milk could achieve comparable outcomes.

The Milk group received the AIN-93M diet supplemented with 242 g/kg FA- and CBP-fortified milk powder (NUTRIPOWER^®^ kids milk powder), supplied by Mead Johnson Nutrition and Health Innovation Institute (Guangzhou, China). The fortified diet provided 47.88 μg folic acid/100 g and 24.20 mg CBP/100 g. This FA concentration corresponds to ~114–171 μg/day for children consuming 2–3 servings (30 g/serving), approximating the Chinese RNI for children ≥4 years (190 μg/day) [23]. For CBP, previous studies found 0.15% effective for bone enhancement, whereas 0.015% was ineffective [24]. Therefore, 0.1% (*w/w*) CBP was selected as a biologically active and translationally appropriate dose.

All diets were isocaloric (3.85–3.90 kcal/g) to ensure that observed differences were not attributable to energy intake.

### 2.3. Data Collection

Body weight, total body length (from the tip of the nose to the tip of the tail), and food intake were recorded twice per week throughout the 8-week intervention.

Body length was measured by two trained experimenters for accuracy and reproducibility: one held the mouse in a natural, fully extended position, while the other measured the distance from the nose to the tail tip using a digital caliper. All routine measurements were performed on conscious animals to minimize stress. For the terminal measurements at intervention week 4 and week 8, body length was measured under light anesthesia immediately before sacrifice.

At intervention weeks 4 and 8, 20 mice per group (10 males, 10 females) were euthanized using sodium pentobarbital (150 mg/kg, i.p.). Blood was collected by cardiac puncture, centrifuged at 3000 rpm for 10 min at 4 °C (Eppendorf 5810R, Hamburg, Germany), and serum samples were stored at −80 °C for biochemical analysis.

Hazard statement: Sodium pentobarbital was used for euthanasia. This compound and paraformaldehyde are hazardous and were handled in accordance with institutional biosafety regulations.

### 2.4. Micro-Computed Tomography (Micro-CT) Analysis

Left femurs were dissected, cleaned, and fixed in 4% paraformaldehyde for 24 h. Bone microstructure was assessed using micro-CT (Skyscan 1176, Bruker, Kontich, Belgium) at 50 kV, 200 μA, 8.7 μm voxel resolution. Reconstruction was performed with NRecon v1.7.3.0 (Bruker, Kontich, Belgium), with beam hardening correction (30%) and ring artifact reduction (5%). Alignment was confirmed in three planes using DataViewer v1.5.6 (Bruker, Kontich, Belgium).

Trabecular bone was analyzed in a 2 mm region of interest (ROI) immediately distal to the growth plate, and cortical bone was analyzed in a 0.5 mm midshaft ROI. A global threshold of 60–255 grayscale units was applied to segment mineralized tissue, following manufacturer-recommended calibration. Morphometric indices were quantified using CTAn v1.16 (Bruker, Kontich, Belgium) and included bone volume fraction (BV/TV), volumetric bone mineral density (vBMD), trabecular thickness (Tb.Th), number (Tb.N), separation (Tb.Sp), structure model index (SMI), trabecular pattern factor (Tb.Pf), bone surface-to-volume ratio (BS/BV), and bone surface density (BS/TV).

### 2.5. Histology Analysis

#### 2.5.1. Decalcification and Sectioning

Right femurs and tibiae were fixed in 4% paraformaldehyde for 24 h, then decalcified in 10% ethylenediaminetetraacetic acid (EDTA, pH 7.4) at 4 °C for 14 days with gentle agitation. The decalcifying solution was refreshed every 2–3 days. Samples were embedded in paraffin and sectioned at 5-μm thickness for subsequent staining.

#### 2.5.2. TRAP Staining Procedure

Tartrate-resistant acid phosphatase (TRAP) staining was performed using the Sigma-Aldrich kit (Cat. 387A, Sigma-Aldrich, St. Louis, MO, USA). TRAP-positive multinucleated cells (>3 nuclei) were counted in three fields per section (100×). TRAP-positive area fraction was quantified using ImageJ version 1.53e (Wayne Rasband and contributors, National Institutes of Health, Bethesda, MD, USA; http://imagej.nih.gov/ij) 

#### 2.5.3. Safranin O/Fast Green Staining

Sections were stained with 0.1% Fast Green FCF (pH 4.2) and 0.1% Safranin O (pH 2.0). Cartilage proteoglycan (red) and mineralized bone (green) were quantified in three fields per section at 40× and 100×. Safranin O-positive ratio was calculated as Red/(Red + Green) using ImageJ.

### 2.6. Measurement of Serum Bone Turnover Markers

Serum osteocalcin (OCN) and C-terminal telopeptide of type I collagen (CTX-I) were measured using commercial ELISA kits (Zokeyo Biotechnology, Wuhan, China; Cat. No. SH32994 and Y-100673). All assays were performed in duplicate; inter-/intra-assay CV < 10%. Absorbance was read at 450 nm on Tecan Infinite 200 Pro, with standard curve R^2^ ≥ 0.99.

### 2.7. GH–IGF-1 Axis Indicators

Serum growth hormone (GH), insulin-like growth factor 1 (IGF-1), and IGF-binding protein 3 (IGFBP-3) were measured via ELISA (Zokeyo Biotechnology, Wuhan, China;; Cat. SH33218, SH33314, SH33324). All assays were performed in duplicate, with inter- and intra-assay CVs < 10%. Absorbance was read at 450 nm using a Tecan Infinite 200 Pro microplate reader (Tecan Group Ltd., Männedorf, Switzerland), with R^2^ ≥ 0.99 for standard curves.

### 2.8. Glucose and Lipid Metabolism Indicators

Serum glucose, total cholesterol (TC), triglycerides (TG), high-density lipoprotein cholesterol (HDL-C), and low-density lipoprotein cholesterol (LDL-C) were quantified using commercial enzymatic colorimetric kits (Beikong Biotechnology, Beijing, China). Glucose was measured by the glucose oxidase–peroxidase (GOD–POD) method; TC by CHOD–PAP; TG by GPO–PAP; and HDL-C and LDL-C by direct enzymatic assays. All blood samples were collected after an overnight fast (12 h) to ensure baseline metabolic levels. All assays were performed in duplicate.

### 2.9. Statistical Analysis

Statistical analyses were performed using SPSS 26.0 (IBM, Armonk, NY, USA), and figures were generated with GraphPad Prism 9.0.1 (GraphPad Software, San Diego, CA, USA). Sample size was based on prior juvenile mouse studies to yield 80% power (α = 0.05).

Longitudinal data (body weight, length) were analyzed using two-way repeated-measures ANOVA, with time as the within-subject factor and group as the between-subject factor, followed by Bonferroni-adjusted post hoc tests.

Endpoint variables were analyzed by one-way ANOVA or Kruskal–Wallis test, followed by Tukey’s or Bonferroni post hoc tests as appropriate, with all pairwise comparisons adjusted for multiple testing.

Sex-specific effects were evaluated using two-way ANOVA (sex × group).

Data are expressed as mean ± SD, and *p* < 0.05 was considered statistically significant (two-sided).

## 3. Results

### 3.1. Effects on Linear Growth

**Figure 1:**
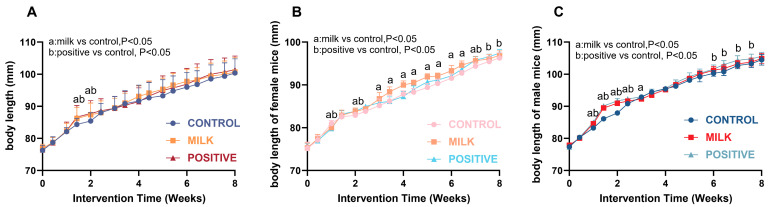
Growth curves of body length in mice. (**A**) Growth curves for all mice. (**B**) Growth curves for female mice. (**C**) Growth curves for male mice. Data are presented as mean ± SD. Statistical analyses were performed using two-way repeated-measures ANOVA (time × group) followed by Bonferroni post hoc tests. a, *p* < 0.05 for Milk vs. Control; b, *p* < 0.05 for Positive Control vs. Control. Sample size: n = 40 per group (20 males + 20 females) for intervention weeks 0–4; n = 20 per group (10 males + 10 females) for intervention weeks 4–8.

Food intake was comparable across groups throughout the 8-week intervention (Figure S1), and no gross abnormalities were observed upon dissection. Body length increased in a biphasic pattern, with rapid early growth followed by attenuation (Figure 1A; Table S3). At intervention week 2, corresponding to the juvenile–adolescent transition, the Milk group exhibited a transient, statistically significant increase in growth velocity compared with the Control group. In females, it was 83.78 ± 0.82 mm, and 82.91 ± 0.71 mm for the Milk and Control group, respectively (*p* < 0.01; Figure 1B; Table S4). In males, body length was 90.96 ± 0.75 mm in the Milk group and 87.90 ± 0.56 mm in Control group (*p* < 0.001; Figure 1C; Table S5). By intervention week 8, mean body length did not differ significantly between the Milk and Control groups in either sex (all *p* > 0.05).

### 3.2. Micro-CT Results of Femurs

**Figure 2:**
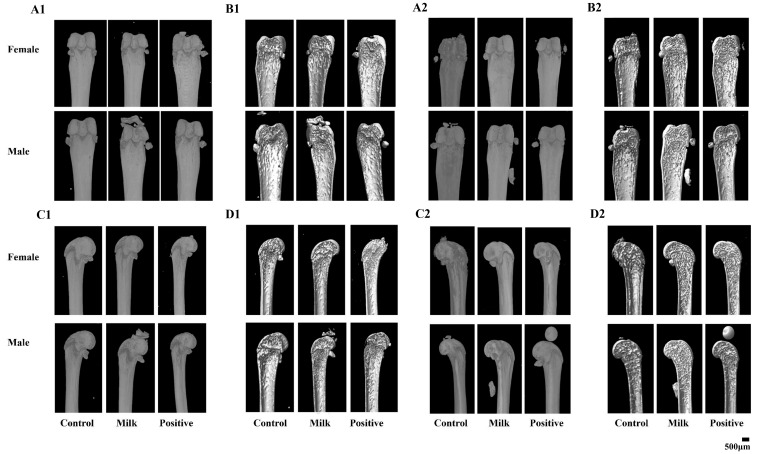
Representative micro-CT images of femoral microarchitecture following 4 and 8 weeks of intervention. (**A1**,**A2**) Surface 3D overviews at 4 weeks (**A1**) and 8 weeks (**A2**) (direct surface visualization). (**B1**,**B2**) Virtual mid-sagittal sections (virtual cut through the mid-diaphysis) showing internal trabecular and cortical architecture at 4 weeks (**B1**) and 8 weeks (**B2**). (**C1**,**C2**) Coronal 3D overviews at 4 weeks (**C1**) and 8 weeks (**C2**). (**D1**,**D2**) Coronal cross-sectional views at 4 weeks (**D1**) and 8 weeks (**D2**). A single 500 μm scale bar is shown in the bottom-right corner of the composite figure and applies to all panels.

Representative micro-CT images showed apparent improvements in trabecular architecture in the Milk and Positive Control groups compared with Control (Figure 2). Quantitative analysis confirmed these differences for parameters reaching statistical significance (Table 1). At intervention week 4, male mice in the Milk group exhibited higher BV/TV (5.59 ± 0.56% vs. 4.86 ± 0.94%, *p* < 0.01) and lower Tb.Pf (21.8 ± 0.96 vs. 24.6 ± 1.56 mm^−1^, *p* < 0.01) than the Control. Positive controls showed the highest BV/TV (7.32 ± 1.38%, *p* < 0.05 vs. Milk), BS/TV (4.29 ± 0.73 mm^−1^, *p* < 0.05 vs. Milk) and Tb.N (1.02 ± 0.20 mm^−1^, *p* < 0.05 vs. Milk).

At intervention week 8, male mice receiving Milk powder showed statistically significant improvements in bone parameters compared with the Control: BV/TV (25.10 ± 5.83% vs. 15.00 ± 3.38%, *p* < 0.01), BS/TV (12.20 ± 1.43 vs. 5.51 ± 2.44 mm^−1^, *p* < 0.01), Tb.N (3.04 ± 0.57 vs. 1.33 ± 0.70 mm^−1^, *p* < 0.01), Tb.Pf (10.80 ± 4.35 vs. 24.70 ± 1.24 mm^−1^, *p* < 0.01), SMI (1.44 ± 0.49 vs. 2.22 ± 0.44, *p* < 0.05), and Tb.Sp (0.23 ± 0.04 vs. 0.55 ± 0.18 mm, *p* < 0.01). Compared with the Positive Control, Milk-treated males had higher BV/TV and Tb.N and lower Tb.Pf, SMI, and Tb.Sp (all *p* < 0.05). In females at week 8, Milk supplementation resulted in a statistically significant increase in BS/TV relative to the Control (4.45 ± 1.87 vs. 2.97 ± 0.59 mm^−1^, *p* < 0.05). The Positive Control group showed the largest increases across the measured trabecular parameters.

### 3.3. Histological Assessment of Bone Morphology

#### 3.3.1. TRAP Staining

**Figure 3:**
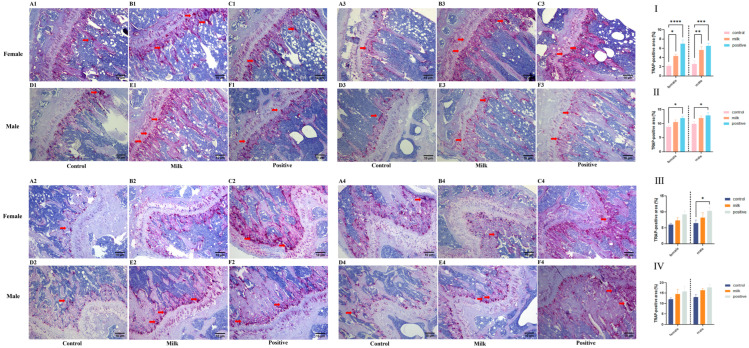
Osteoclast activity as assessed by TRAP staining (representative images and quantification). Representative photomicrographs of TRAP-stained sections (red indicates TRAP-positive osteoclasts) from female and male mice after 4 weeks (Rows 1,2) and 8 weeks (Rows 3,4) of intervention. Red indicates TRAP-positive osteoclasts (red arrows). (**A1**–**F2**, 4-week): Tibiae (**A1**–**C1**: females; **D1**–**F1**: males) and femurs (**A2**–**C2**: females; **D2**–**F2**: males). (**A3**–**F4**, 8-week): Tibiae (**A3**–**C3**: females; **D3**–**F3**: males) and femurs (**A4**–**C4**: females; **D4**–**F4**: males). The bar graphs (**I**–**IV**) quantify the TRAP-positive area fraction (%): (**I**) femurs after the 4-week intervention; (**II**) tibiae after the 4-week intervention; (**III**) femurs after the 8-week intervention; (**IV**) tibiae after the 8-week intervention. Data for female (♀, left) and male (♂, right) mice are presented as individual data points with mean (n = 6 per sex per group). * *p* < 0.05, ** *p* < 0.01, *** *p* < 0.001, **** *p* < 0.0001 vs. Control.

TRAP staining demonstrated group- and time-dependent differences in osteoclast activity (Figure 3). At intervention week 4, the TRAP-positive area fraction in femurs was significantly increased in both males and females in the Milk and Positive Control groups compared with Control (*p* < 0.05; Figure 3I). In tibiae, significant increases were observed only in the Positive Control group for both sexes (both *p* < 0.05; Figure 3II).

At intervention week 8, a significant increase was detected only in male femurs of the Positive Control group, while female femurs showed no significant group differences (Figure 3III). No significant changes were observed in tibiae of either sex at intervention week 8 (Figure 3IV).

#### 3.3.2. Safranin O-Fast Green Staining

**Figure 4:**
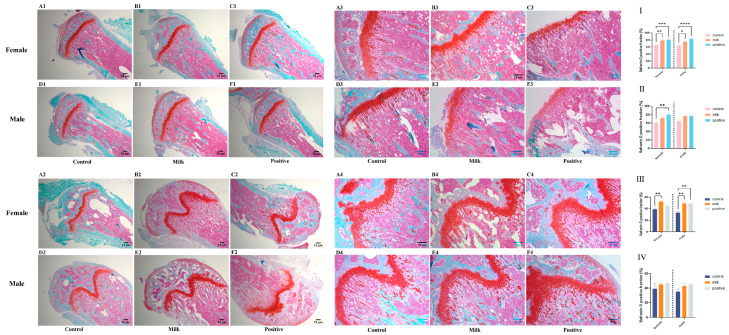
Bone and cartilage matrix composition by Safranin O/Fast Green staining (representative images and quantification). Representative photomicrographs of stained sections (Safranin O red indicates cartilage proteoglycans; Fast Green indicates mineralized bone) from female and male mice after 4 weeks (Rows 1,2) and 8 weeks (Rows 3,4) of intervention. (**A1**–**F2**, 4-week): Tibiae (**A1**–**C1**: females; **D1**–**F1**: males) and femurs (**A2**–**C2**: females; **D2**–**F2**: males). (**A3**–**F4**, 8-week): Tibiae (**A3**–**C3**: females; **D3**–**F3**: males) and femurs (**A4**–**C4**: females; **D4**–**F4**: males). The bar graphs (**I**–**IV**) quantify the Safranin O-positive area ratio [Red/(Red + Green), %]: (**I**) femurs after the 4-week intervention; (**II**) tibiae after the 4-week intervention; (**III**) femurs after the 8-week intervention; (**IV**) tibiae after the 8-week intervention. Data for female (♀, left) and male (♂, right) mice are presented as individual data points with mean (n = 6 per sex per group). * *p* < 0.05, ** *p* < 0.01, *** *p* < 0.001, **** *p* < 0.0001 vs. Control.

Safranin O–Fast Green staining indicated treatment- and time-dependent changes in bone mineralization (Figure 4). At intervention week 4, femurs of both male and female mice in the Milk and Positive Control groups exhibited significantly higher Safranin O–positive ratios [Red/(Red + Green)] compared with Control (*p* < 0.05; Figure 4I). In the tibiae, a significant increase was observed only in females of the Positive Control group (*p* < 0.01; Figure 4II).

At intervention week 8, both male and female femurs in the Milk group showed significantly higher Safranin O–positive ratios compared with Controls (*p* < 0.05; Figure 4III). No significant changes were observed in the tibiae of either sex (Figure 4IV).

### 3.4. Serum Bone Turnover Markers

**Figure 5:**
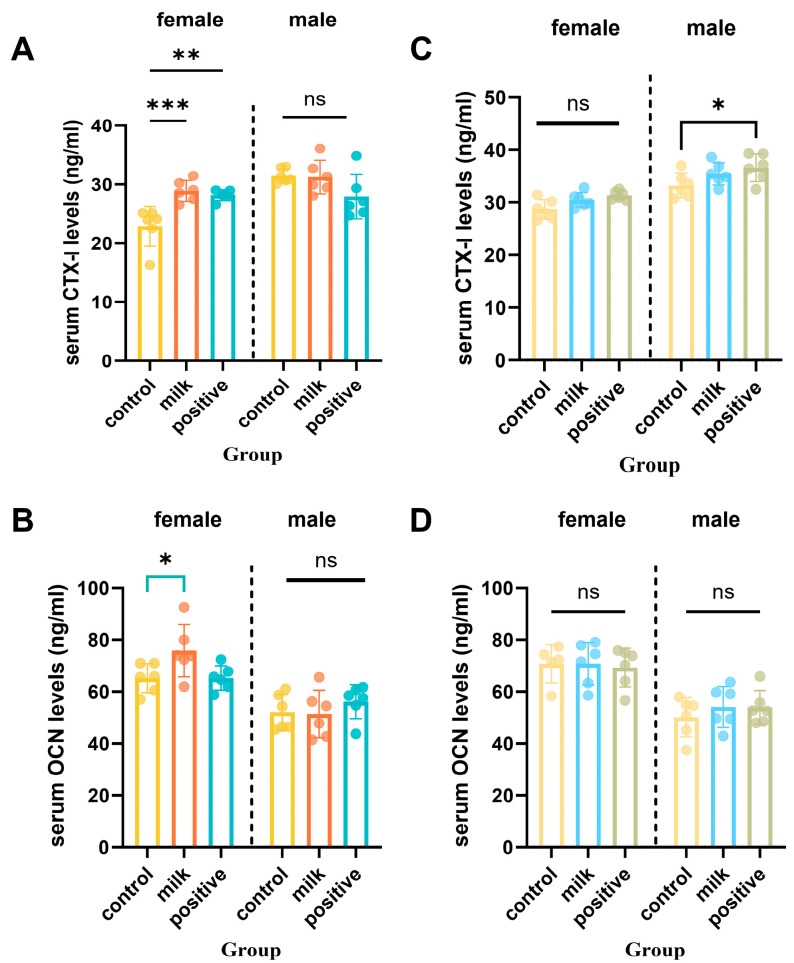
Serum bone resorption (CTX-I) and formation (OCN) biomarkers after 4 and 8 intervention weeks. (**A**,**B**) Serum levels of CTX-I (C-terminal telopeptide of type I collagen) after 4 and 8 intervention weeks. (**C**,**D**) Serum levels of osteocalcin (OCN) after 4 and 8 intervention weeks, respectively. Data are shown separately for females (left panels) and males (right panels). Values are presented as individual data points with mean (n = 6 per sex per group). * *p* < 0.05, ** *p* < 0.01, *** *p* < 0.001 vs. Control, ns: not significant.

Serum bone turnover markers exhibited sex- and time-specific changes (Figure 5). At intervention week 4, female mice in the Milk group had significantly higher serum CTX-I (*p* < 0.01) and osteocalcin (*p* < 0.05) than Control group, whereas males showed no significant differences. By intervention week 8, Milk and Control groups showed comparable CTX-I and osteocalcin levels in both sexes (*p* > 0.05). In males, the Positive Control group exhibited significantly higher CTX-I and osteocalcin compared with Controls (*p* < 0.05).

### 3.5. Growth Hormone and IGF-1 Axis

**Figure 6:**
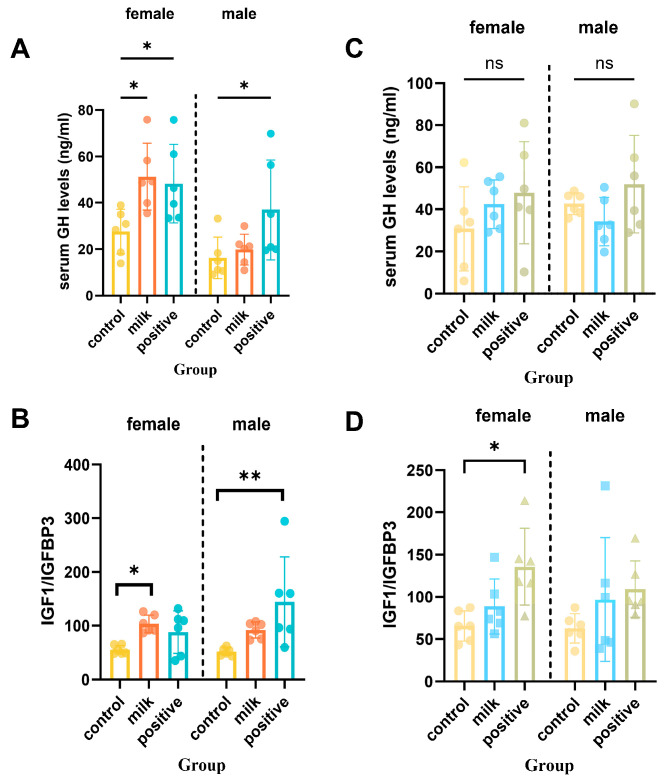
Serum GH and IGF-1 axis markers after 4 and 8 intervention weeks. (**A**) Serum growth hormone (GH) levels after 4 weeks of intervention. (**B**) The IGF-1 to IGFBP-3 molar ratio after 4 weeks of intervention. (**C**) Serum GH levels after 8 intervention weeks. (**D**) The IGF-1 to IGFBP-3 molar ratio after 8 intervention weeks. Data are shown separately for females (left panels) and males (right panels). Values are presented as individual data points with mean (n = 6 per sex per group). * *p* < 0.05, ** *p* < 0.01 vs. Control, ns: not significant.

At intervention week 4, Milk-treated females had elevated serum GH compared with Control (*p* < 0.05), while males showed no significant difference (Figure 6A). Serum IGF-1 in females was not statistically significant (*p* = 0.053), whereas IGFBP-3 levels remained unchanged (Figure S2). To approximate IGF-1 bioavailability, the IGF-1/IGFBP-3 molar ratio was calculated and this ratio was significantly higher in Milk females than Controls at intervention week 4 (*p* < 0.05; Figure 6B). By intervention week 8, GH, IGF-1, and IGF-1/IGFBP-3 ratios remained significantly elevated in females of the Milk group compared with Controls (*p* < 0.05), whereas no significant differences were observed among males (*p* > 0.05).

### 3.6. Effects on Body Weight and Metabolic Parameters

#### 3.6.1. Body Weight

**Figure 7:**
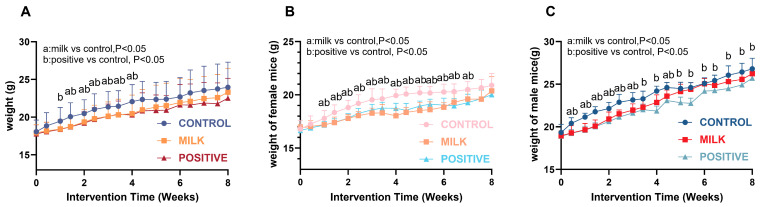
Body weight changes in mice during intervention. (**A**) Weight curves for all mice. (**B**) Weight curves for female mice. (**C**) Weight curves for male mice. Data are presented as mean ± SD. Statistical significance is indicated as: a, *p* < 0.05 for Milk vs. Control; b, *p* < 0.05 for Positive Control vs. Control.

All groups showed progressive weight gain during the 8-week intervention (Figure 7A). Final body weight did not differ between the Milk and Control groups (*p* > 0.05). In females, both the Milk and Positive Control groups exhibited slower weight gain than the Control during intervention weeks 2–6 (*p* < 0.05), and final weights were comparable across all groups (Figure 7B). In males, the Positive Control group had lower body weight than the Control from intervention week 1 onward (*p* < 0.05). The Milk group showed lower body weight than the Control during intervention weeks 1–3, and no significant differences were observed during weeks 5–8 (Figure 7C).

#### 3.6.2. Fasting Blood Glucose

**Figure 8:**
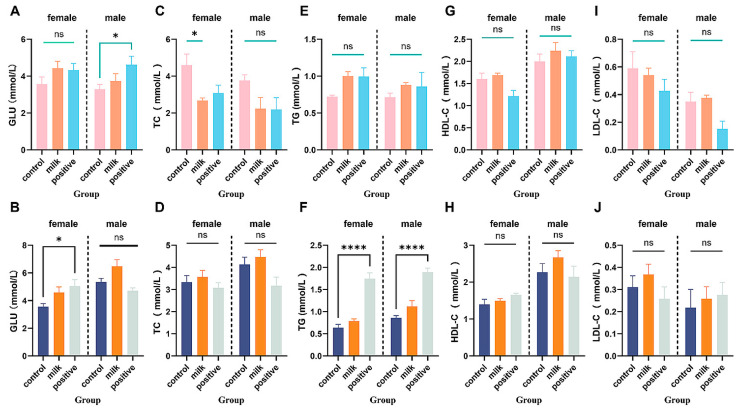
Fasting blood glucose and serum lipid levels in all groups. (**A**,**B**) Fasting blood glucose levels at 4 and 8 intervention weeks, respectively. (**C**–**J**) Serum lipid parameters: TC (**C**,**D**), TG (**E**,**F**), LDL-C (**G**,**H**), HDL-C (**I**,**J**). Data are shown separately for females (left panels) and males (right panels). Values are expressed as mean ± SD (n = 6 per sex per group). * *p* < 0.05, **** *p* < 0.0001 vs. Control. ns: not significant.

Milk supplementation did not affect fasting blood glucose at 4 or 8 intervention weeks in either sex (both *p* > 0.05; Figure 8A,B). The Positive Control group showed sex-specific effects: elevated fasting blood glucose in males at intervention week 4 (*p* < 0.05) and in females at intervention week 8 (*p* < 0.05).

#### 3.6.3. Serum Lipid Parameters

At intervention week 4, Milk-supplemented females had reduced total cholesterol (TC; *p* < 0.05) with no changes in TG, LDL-C, or HDL-C; all parameters were comparable to the Control by intervention week 8 (Figure 8C–J)**.** The Positive Control diet elicited several parameter-specific perturbations, including increased TG in both sexes (*p* < 0.001).

## 4. Discussion

This study demonstrates that FA–CBP-fortified milk supplementation enhanced skeletal development, characterized by accelerated linear growth during puberty and improved trabecular bone microarchitecture in early adulthood. Importantly, these benefits were achieved without detectable adverse effects on body weight or metabolic profiles, supporting the safety of this nutritional approach in growing mice. Given the importance of achieving optimal bone accrual during youth, these findings highlight FA–CBP-fortified milk as a potentially effective strategy to support healthy skeletal growth.

Micro-CT analysis revealed that FA–CBP supplementation led to measurable improvements in trabecular bone quality, most notably in male mice, who exhibited increases in BV/TV, Tb.N, and trabecular connectivity (reflected by lower SMI) (Figure 2, Table 1). These findings reflect both quantitative and qualitative gains in bone structure and are consistent with epidemiological observations linking inadequate folate status to compromised trabecular structure in humans [25]. Compared with AIN-93G, FA–CBP produced comparable or greater improvements in trabecular structure. This suggests that FA-CBP fortification may exert skeletal benefits that are not solely attributable to macronutrient or calcium supply.

Our data indicate that FA–CBP exerts coordinated effects on systemic endocrine pathways and local bone remodeling. We observed a transient, sex-specific activation of the GH–IGF-1 axis, reflected by early increases in serum GH and a higher IGF-1/IGFBP-3 ratio in females, which likely contributed to the initial acceleration in linear growth (Figure 6). Previous studies have shown that CBP can stimulate GH secretion [11,24], and FA may enhance GH/IGF-1 responsiveness through methylation-dependent transcriptional regulation of pathway components [26,27]. The temporal association between endocrine activation and early growth responses suggests that FA and CBP act synergistically to enhance hormonal signaling during peak developmental windows.

In addition to systemic effects, FA–CBP supplementation was associated with increased local skeletal remodeling. Serum osteocalcin and CTX-I increased during early intervention, indicating enhanced bone turnover (Figure 5). Histology further showed greater osteoclast activity and increased deposition of proteoglycan-rich matrix within the growth plate, consistent with active endochondral ossification (Figure 3 and Figure 4). In males, the combination of elevated remodeling indices and transient reductions in circulating calcium and phosphorus (Figure S3) suggests preferential mineral utilization for skeletal development. This is consistent with the documented capacity of CBP to facilitate mineral utilization and matrix consolidation [28,29], while FA may further support osteoblast function through epigenetic regulation [30], mitigation of homocysteine-induced endoplasmic reticulum stress [31], and activation of AMPK-mediated antioxidant pathways [32]. Together, these findings support a mechanistic model in which FA enhances osteogenic signaling and cellular responsiveness, and CBP supplies substrates and facilitates mineral handling—resulting in coordinated improvements in bone quality.

Sex-specific responses were a prominent feature of our findings. Females exhibited stronger endocrine activation and earlier linear growth acceleration, whereas males demonstrated more pronounced gains in trabecular architecture. This pattern is consistent with established sex differences in skeletal biology: males typically display greater periosteal expansion and higher mineral accretion efficiency [33], and loss of GH receptor signaling in osteoblasts produces more severe deficits in males [34]. In our study, these patterns support the proposal that females primarily leverage an endocrine-driven trajectory of linear growth, whereas males rely on a mineral-optimization model that preferentially enhances bone microarchitecture.

Despite enhanced growth, FA-CBP supplementation did not result in excessive weight gain, insulin resistance (normal fasting blood glucose), or dyslipidemia (Figure 7 and Figure 8). By contrast, the Positive Control group (AIN-93G) exhibited elevated triglycerides in both sexes, underscoring the favorable metabolic profile of FA–CBP supplementation. These observations suggest that FA-CBP promotes targeted skeletal growth without increasing metabolic risk, offering a potential strategy for supporting growth enhancement from metabolic risk in pediatric nutrition.

This study’s strengths include its longitudinal design and the integration of growth, endocrine, histological, and microstructural assessments, providing mechanistic insights into the synergistic effects of FA–CBP supplementation. Limitations include the absence of single-nutrient (FA-only or CBP-only) and milk-only control groups, which prevents definitive attribution of observed effects to either component or the milk matrix. Additional limitations are the use of a single mouse strain, a single supplementation dose, discrete measurement time points, and the potential for measurement variability in body length and other parameters. These factors should be considered when interpreting the findings and planning future studies.

Future studies incorporating single-nutrient and milk-only controls will be essential to delineate the independent versus synergistic contributions of FA and CBP. Investigations should also explore dose-dependent effects, transcriptomic regulation within the growth plate, and temporal patterns of bone remodeling. Moreover, emerging evidence suggests that the microbiota–gut–bone axis plays a significant role in IGF-1 signaling, osteoclast activity, and mineral metabolism [35]. Integrating gut microbial profiling with skeletal assessments may help clarify whether some skeletal benefits of FA–CBP supplementation are mediated through microbiota-dependent mechanisms. These approaches will be critical for evaluating the translational potential of FA–CBP interventions in growing human populations.

## 5. Conclusions

FA- and CBP-fortified milk powder significantly promoted linear growth during puberty and improved bone microarchitecture in juvenile mice, without inducing adverse metabolic effects. These findings indicate that combined FA and CBP fortification may serve as a practical nutritional strategy to support healthy skeletal development during growth while maintaining metabolic stability.

## Figures and Tables

**Table 1:** Femoral bone microstructure parameters in all groups after intervention.

	BV/TV (%)	BS/BV (1/mm)	BS/TV (1/mm)	Tb.Pf (1/mm)	SMI	Tb.Th (mm)	Tb.N (1/mm)	Tb.Sp (mm)
4 Weeks								
All								
Control	3.48 ± 1.64	74.60 ± 13.10	2.42 ± 0.83	27.60 ± 4.33	2.23 ± 0.12	0.06 ± 0.01	0.55 ± 0.19	0.85 ± 0.06
Milk	4.51 ± 1.21	67.90 ± 7.10	3.01 ± 0.69	23.30 ± 3.26	2.17 ± 0.14	0.07 ± 0.01	0.71 ± 0.21	0.81 ± 0.09
Positive Control	5.18 ± 2.63	70.50 ± 13.80	3.34 ± 1.31	25.30 ± 5.58	2.14 ± 0.12	0.06 ± 0.01	0.78 ± 0.32	0.76 ± 0.11
Female								
Control	2.09 ± 0.66	85.10 ± 9.41	1.76 ± 0.51	30.70 ± 4.10	2.16 ± 0.13	0.05 ± 0.01	0.39 ± 0.11	0.89 ± 0.05
Milk	3.74 ± 1.29	71.90 ± 7.50	2.63 ± 0.75	24.80 ± 4.15	2.17 ± 0.10	0.07 ± 0.01	0.64 ± 0.25	0.82 ± 0.11
Positive	3.03 ± 1.53	82.10 ± 8.98	2.39 ± 1.03	29.40 ± 4.83	2.14 ± 0.16	0.05 ± 0.00	0.55 ± 0.25	0.82 ± 0.09
Male								
Control	4.86 ± 0.94 ^b^**	64.20 ± 5.12	3.09 ± 0.44 ^b^**	24.60 ± 1.56 ^b^**	2.30 ± 0.06	0.07 ± 0.01	0.70 ± 0.09 ^b^**	0.81 ± 0.03 ^b^**
Milk	5.59 ± 0.56 ^a^**	63.90 ± 4.06	3.38 ± 0.40	21.80 ± 0.96 ^a^**	2.17 ± 0.18	0.07 ± 0.01	0.78 ± 0.14	0.79 ± 0.06
Positive Control	7.32 ± 1.38 ^c^*	58.90 ± 4.07	4.29 ± 0.73 ^c^*	21.10 ± 1.85	2.15 ± 0.09	0.07 ± 0.01	1.02 ± 0.20 ^c^*	0.70 ± 0.09
8 Weeks								
All								
Control	13.10 ± 3.99 ^b^*	58.40 ± 33.40	4.24 ± 2.15 ^b^**	21.90 ± 4.29 ^b^**	2.37 ± 0.39	0.12 ± 0.05	1.03 ± 0.58 ^b^**	0.66 ± 0.18 ^b^**
Milk	19.80 ± 9.51 ^a^*	57.30 ± 22.20	8.32 ± 4.35 ^a^**	13.90 ± 4.56 ^a^**	1.85 ± 0.56	0.09 ± 0.01 ^a^*	1.97 ± 1.24 ^a^**	0.47 ± 0.30 ^a^**
Positive Control	24.70 ± 5.10 ^c^*	72.60 ± 32.60	10.60 ± 2.72 ^c^*	13.90 ± 1.51	2.25 ± 0.63	0.14 ± 0.06 ^c^*	2.61 ± 0.61 ^c^*	0.33 ± 0.09 ^c^**
All								
Female								
Control	11.20 ± 3.86 ^b^**	55.80 ± 35.90	2.97 ± 0.59 ^b^**	19.00 ± 4.43 ^b^**	2.51 ± 0.30 ^b^*	0.11 ± 0.03	0.73 ± 0.18 ^b^**	0.76 ± 0.13 ^b^**
Milk	11.60 ± 1.69	70.40 ± 25.60	4.45 ± 1.87 ^a^*	17.00 ± 1.97	2.25 ± 0.21	0.08 ± 0.02	0.90 ± 0.56	0.70 ± 0.25
Positive Control	28.00 ± 2.23 ^c^**	73.80 ± 32.70	8.00 ± 1.67 ^c^**	12.60 ± 0.76 ^c^*	1.93 ± 0.46 ^c^*	0.12 ± 0.04	3.02 ± 0.54 ^c^**	0.27 ± 0.06 ^c^**
Male								
Control	15.00 ± 3.38 ^b^**	60.90 ± 33.80	5.51 ± 2.44 ^b^**	24.70 ± 1.24 ^b^**	2.22 ± 0.44	0.12 ± 0.07	1.33 ± 0.70 ^b^**	0.55 ± 0.18 ^b^**
Milk	25.10 ± 5.83 ^a^**	44.20 ± 24.18	12.20 ± 1.43 ^a^**	10.80 ± 4.35 ^a^**	1.44 ± 0.49 ^a^*	0.09 ± 0.01	3.04 ± 0.57 ^a^**	0.23 ± 0.04 ^a^**
Positive Control	21.50 ± 5.10 ^c^*	71.30 ± 35.70	9.25 ± 3.00 ^c^*	15.20 ± 0.70 ^c^*	2.58 ± 0.65 ^c^*	0.16 ± 0.08	2.20 ± 0.36 ^c^*	0.39 ± 0.06 ^c^*

Data are mean ± SD. ^a^: Milk vs. Control; ^b^: Positive Control vs. Control; ^c^: Milk vs. Positive Control. *, *p* < 0.05; **, *p* < 0.01. BV/TV, bone volume fraction; BS/BV, bone surface–to–bone volume ratio; BS/TV, bone surface density; Tb.Pf, trabecular bone pattern factor; SMI, structure model index; Tb.Th, trabecular thickness; Tb.N, trabecular number; Tb.Sp, trabecular separation.

## Data Availability

The original contributions presented in this study are included in the article/Supplementary Material. Further inquiries can be directed to the corresponding author.

## Supplementary Materials

The following supporting information can be downloaded at: https://www.mdpi.com/article/10.3390/nu17243819/s1.
