# Supplementary material for: Milk Powder Fortified with Folic Acid and Colostrum Basic Protein Promotes Linear Growth and Improves Bone Microarchitecture in Juvenile Mice Without Adverse Metabolic Effects
Source: Nutrients. 2025 Dec 5;17(24):3819. doi: 10.3390/nu17243819 (PMC12736383; doi:10.3390/nu17243819)
Supplement: Supplementary file 1 [file nutrients-17-03819-s001.zip › nutrients-3966636-supplementary.pdf]

Milk Powder Fortified with Folic Acid and Colostrum Basic Protein Promotes Linear Growth and Improves Bone Microarchitecture in Juvenile Mice Without Adverse Metabolic Effects

Hongjuan Liu[#1, 2], Yixin Zhang[#1], Yuanjue Wu[1], Shuoye Tan[1], Zhe Zhang[1], Wenbo Wan[3], Jiawen Liang[3], Liping Hao[2], Ting Xiong[*1]

[1] Department of Nutrition and Food Hygiene, School of Public Health, Guangzhou Medical University, Xinzao, Panyu District, Guangzhou, 511436, P.R.China.

[2] Department of Nutrition and Food Hygiene, Hubei Key Laboratory of Food Nutrition and Safety and the Ministry of Education (MOE) Key Laboratory of Environment and Health, School of Public Health, Tongji Medical College, Huazhong University of Science and Technology, 13 Hangkong Rd, Wuhan, 430030, Hubei, China.

[3] Mead Johnson Nutrition and Health Innovation Institute, No. 28, Zhujiang Road East, Zhujiang New Town, Guangzhou, 510623, P.R.China.

Table S1. Dietary compositions for each group

Nutrients	Unit	Control	Milk	Positive
**Energy**	kcal/g	3.85	3.90	3.90
**Protein**	g/100 g	14.20	15.48	20.00
**Carbohydrate**	g/100 g	73.10	68.99	64.00
**Fat**	g/100 g	4.00	6.25	7.00
**Vitamin A**	IU/100 g	400.00	654.58	400.00
**Vitamin D3**	IU/100 g	100.00	133.88	100.00
**Vitamin E**	IU/100 g	7.50	7.50	7.50
**Biotin**	mg/100 g	0.08	0.06	0.08
**Vitamin B12**	mg/100 g	0.02	0.02	0.02
**Folic acid**	μg/100 g	2.50	47.88	2.50
**Niacin**	mg/100 g	0.20	1.00	0.20
**Pantothenic acid**	mg/100 g	3.00	3.25	3.00
**Vitamin B6**	mg/100 g	1.60	1.32	1.60
**Vitamin B2**	mg/100 g	0.70	0.75	0.70
**Vitamin B1**	mg/100 g	0.60	0.64	0.60
**Calcium (Ca)**	g/100 g	0.50	0.67	0.50
**Phosphorus (P)**	g/100 g	0.20	0.25	0.15
**Potassium (K)**	g/100 g	0.36	0.27	0.36
**Sulfur (S)**	g/100 g	0.03	0.02	0.03
**Magnesium (Mg)**	g/100 g	0.05	0.06	0.05
**Sodium (Na)**	g/100 g	0.10	0.08	0.10
**Chloride (Cl)**	g/100 g	0.16	0.12	0.16
**Copper (Cu)**	mg/100 g	0.60	0.53	0.60
**Iodine (I)**	mg/100 g	0.02	0.02	0.02
**Iron (Fe)**	mg/100 g	4.50	5.61	3.70
**Manganese (Mn)**	mg/100 g	1.05	0.97	1.05
**Selenium (Se)**	mg/100 g	0.02	0.02	0.02

**Zinc (Zn)**	mg/100 g	3.00	3.86	3.00
**Chromium (Cr)**	mg/100 g	0.10	0.07	0.10
**Molybdenum (Mo)**	mg/100 g	0.02	0.01	0.02
**Fluoride (F)**	mg/100 g	0.10	0.08	0.10
**Casein phosphopeptides (CBP)**	mg/100 g	—	24.2	—

Footnote: “—” indicates not added.

Table S2. Amino acid composition of the experimental diets (mg/100 g diet)

Amino acid	Unit	Control	Positive	Milk
Aspartate	mg/100 g	864.78	1218.00	1040.06
Threonine	mg/100 g	545.28	768.00	635.82
Serine	mg/100 g	719.94	1014.00	821.41
Glutamate	mg/100 g	2976.32	4192.00	3329.78
Glycine	mg/100 g	232.88	328.00	273.26
Alanine	mg/100 g	397.60	560.00	470.68
Valine	mg/100 g	815.08	1148.00	944.32
Isoleucine	mg/100 g	661.72	932.00	770.04
Leucine	mg/100 g	1218.36	1716.00	1419.31
Tyrosine	mg/100 g	763.96	1076.00	816.05
Phenylalanine	mg/100 g	653.20	920.00	739.38
Lysine	mg/100 g	1015.30	1430.00	1185.58
Histidine	mg/100 g	360.68	508.00	449.96
Arginine	mg/100 g	475.70	670.00	529.86
Proline	mg/100 g	1371.72	1932.00	1528.26
Methionine	mg/100 g	366.36	516.00	408.29

Table S3. Body length changes during the intervention period

intervention time	control	milk	positive	p-value
**Week 0**	76.27 ± 1.56	76.92 ± 1.25	76.42 ± 1.09	0.070
**Week 0.5**	78.70 ± 1.80	78.74 ± 1.56	78.62 ± 1.73	0.951
**Week 1**	82.11 ± 1.23	82.32 ± 2.40	82.40 ± 2.68	0.829
**Week 1.5**	84.34 ± 1.92	86.20 ± 3.43*	86.65 ± 3.55**	0.002
**Week 2**	85.40 ± 2.57	87.37 ± 3.65*	87.75 ± 4.00**	0.006
**Week 2.5**	88.03 ± 2.87	88.01 ± 3.66	88.62 ± 3.73	0.666
**Week 3**	89.41 ± 3.68	89.52 ± 2.98	89.39 ± 3.67	0.984
**Week 3.5**	90.83 ± 4.03	91.02 ± 3.07	90.33 ± 4.28	0.706
**Week 4**	90.65 ± 4.92	93.08 ± 2.29	91.43 ± 4.31	0.106
**Week 4.5**	91.66 ± 4.75	94.13 ± 2.74	93.17 ± 4.34	0.110
**Week 5**	93.28 ± 5.00	95.27 ± 3.51	94.98 ± 4.46	0.239
**Week 5.5**	94.78 ± 4.68	96.58 ± 3.68	95.72 ± 4.80	0.376
**Week 6**	95.97 ± 4.56	97.76 ± 3.71	96.87 ± 4.96	0.383
**Week 6.5**	96.83 ± 4.24	98.21 ± 3.82	98.34 ± 4.88	0.410
**Week 7**	98.55 ± 4.36	99.43 ± 4.02	100.06 ± 4.51	0.478
**Week 7.5**	99.39 ± 4.17	99.97 ± 4.14	100.70 ± 4.34	0.562
**Week 8**	100.38 ± 4.41	100.70 ± 4.46	101.48 ± 4.25	0.672

*$p < 0.05$ vs. Control; ** $p < 0.01$ vs. Control; ** *$p < 0.001$ vs. Control.

Table S4. Body length changes in female rats during the intervention period

intervention time	control	milk	positive	p-value
**Week 0**	75.15 ± 1.49	75.30 ± 1.14	75.54 ± 0.82	0.349
**Week 0.5**	77.08 ± 1.01	77.40 ± 1.04	76.94 ± 0.31	0.216
**Week 1**	80.95 ± 0.71	80.05 ± 0.98**	79.79 ± 0.54***	0.000
**Week 1.5**	82.55 ± 0.72	82.92 ± 1.20	83.19 ± 0.76	0.096
**Week 2**	82.91 ± 0.71	83.78 ± 0.82**	83.82 ± 0.71***	0.000
**Week 2.5**	83.85 ± 0.59	84.41 ± 0.73	84.97 ± 0.58	0.000
**Week 3**	85.15 ± 1.53	86.77 ± 1.47**	85.85 ± 1.05	0.075
**Week 3.5**	86.21 ± 1.33	88.39 ± 1.14***	86.17 ± 1.04	0.002
**Week 4**	87.86 ± 0.66	89.97 ± 0.97***	87.30 ± 1.16	0.000
**Week 4.5**	88.32 ± 0.76	90.52 ± 0.62***	88.97 ± 0.83	0.000
**Week 5**	89.40 ± 0.84	91.89 ± 0.72**	90.70 ± 0.93	0.000
**Week 5.5**	90.34 ± 0.96	92.07 ± 0.70**	91.14 ± 0.67	0.000
**Week 6**	91.57 ± 0.97	93.31 ± 1.22**	92.12 ± 0.79	0.000
**Week 6.5**	92.77 ± 0.60	94.62 ± 0.96***	93.67 ± 0.85	0.000
**Week 7**	94.39 ± 0.95	95.64 ± 1.05*	95.78 ± 0.80**	0.001
**Week 7.5**	95.47 ± 1.08	96.07 ± 1.06	96.59 ± 0.60*	0.022
**Week 8**	96.29 ± 1.19	96.93 ± 1.04	97.44 ± 0.86*	0.018

*$p < 0.05$ vs. Control; ** $p < 0.01$ vs. Control; ** *$p < 0.001$ vs. Control.

Table S5. Body length changes in male rats during the intervention period

intervention time	control	milk	positive	p-value
**Week 0**	77.39 ± 0.34	77.84 ± 0.39	77.31 ± 0.31	0.081
**Week 0.5**	80.32 ± 0.37	80.08 ± 0.45	80.31 ± 0.46	0.051
**Week 1**	83.26 ± 0.45	84.58 ± 0.56***	85.01 ± 0.41***	0.000
**Week 1.5**	86.13 ± 0.51	89.48 ± 0.35***	90.11 ± 0.71***	0.000
**Week 2**	87.90 ± 0.56	90.96 ± 0.75***	91.69 ± 0.83***	0.000
**Week 2.5**	90.81 ± 0.61	91.62 ± 0.52***	92.28 ± 0.65***	0.000
**Week 3**	92.87 ± 0.76	92.28 ± 0.73	93.94 ± 0.75	0.000
**Week 3.5**	94.46 ± 0.57	93.66 ± 0.91	94.49 ± 0.88	0.000
**Week 4**	95.43 ± 0.74	95.19 ± 0.60	95.56 ± 0.61	0.276
**Week 4.5**	96.29 ± 0.42	96.73 ± 0.71	97.37 ± 0.83	0.002
**Week 5**	98.15 ± 0.56	98.64 ± 0.91	99.27 ± 1.15	0.010
**Week 5.5**	99.23 ± 1.62	100.09 ± 1.00	100.31 ± 1.35	0.132
**Week 6**	100.37 ± 0.93	101.21 ± 1.16	101.61 ± 1.30*	0.036
**Week 6.5**	100.88 ± 1.21	101.81 ± 1.17	103.01 ± 1.21***	0.001
**Week 7**	102.71 ± 1.00	103.22 ± 1.13	104.33 ± 1.45**	0.008
**Week 7.5**	103.32 ± 1.23	103.87 ± 1.24	104.82 ± 1.44*	0.028
**Week 8**	104.28 ± 1.64	104.88 ± 1.56	105.52 ± 1.32	0.249

*$p < 0.05$ vs. Control; ** $p < 0.01$ vs. Control; ** *$p < 0.001$ vs. Control.

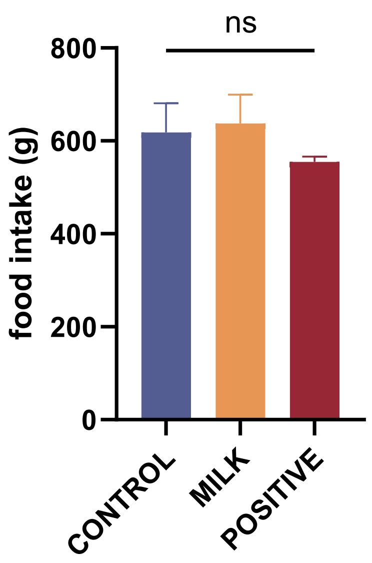

Figure S1. Total Food Intake of All Groups of Mice During the Experimental Period

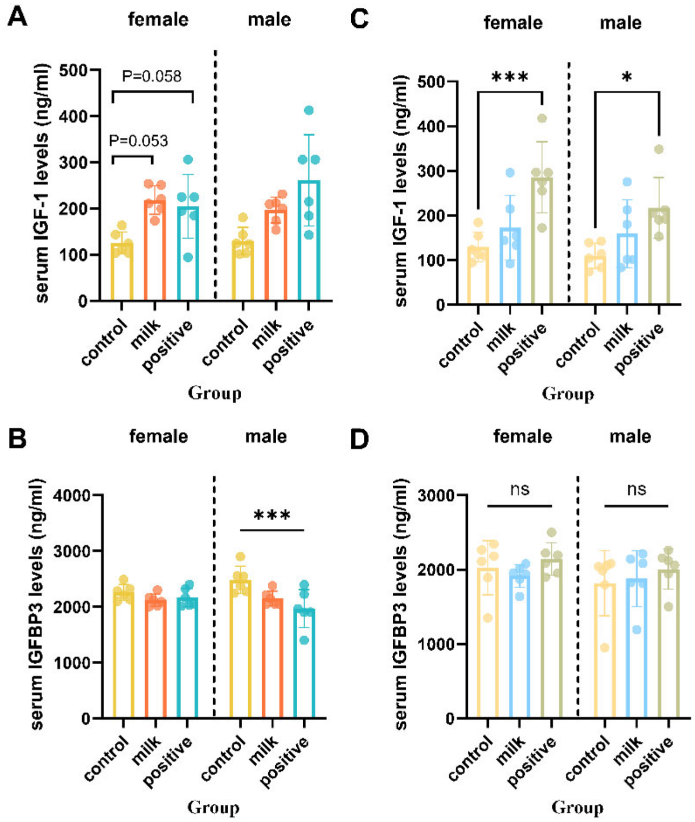

Figure S2. Serum GH and IGF-1 Levels Across Experimental Groups

Values are presented as mean ± SEM with individual data points (n = 6 per sex per group).

Panels:

Figure S2A: Serum IGF-1 levels after 4-week intervention in female and male mice in female (♀, left) and male (♂, right) mice.

Figure S2B: Serum IGF-binding protein 3 (IGFBP3) levels after 4-week intervention in female (♀, left) and male (♂, right) mice.

Figure S2C: Serum IGF-1 levels after 8-week intervention in female (♀, left) and male (♂, right) mice.

Figure S2D: Serum IGFBP3 levels after 8-week intervention in female (♀, left) and male (♂, right) mice.

Each panel displays three experimental groups: Control, FA+CBP Milk, and Positive Control. Female and male mice are presented separately to highlight sex-specific responses. Individual data points are overlaid on bar graphs to illustrate variability within each sex. Statistical significance versus the Control group is indicated by asterisks (*$p< 0.05$; ** $p < 0.01$; *** $p < 0.001$ vs. Control, ns: not significant.).

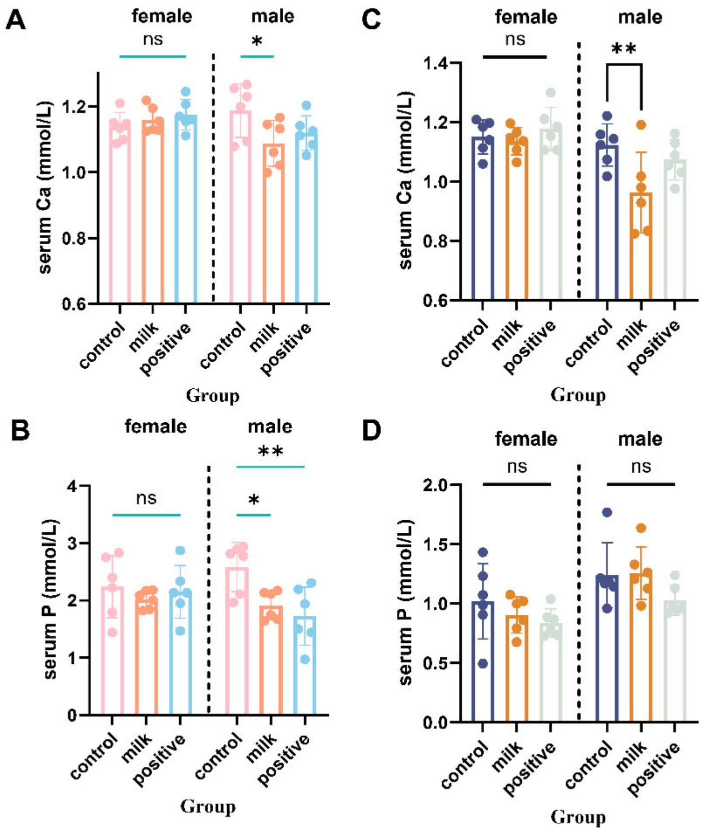

Figure S3. Serum Calcium (Ca) and Phosphorus (P) Levels Across Experimental Groups

Values are presented as mean ± SEM with individual data points (n = 6 per sex per group).
Panels:
Figure S3A: Serum calcium (Ca) levels after 4-week intervention in female (♀, left) and male (♂, right) mice.
Figure S3B: Serum phosphorus (P) levels after 4-week intervention in female (♀, left) and male (♂, right) mice.
Figure S3C: Serum calcium (Ca) levels after 8-week intervention in female (♀, left) and male (♂, right) mice.
Figure S3D: Serum phosphorus (P) levels after 8-week intervention in female (♀, left) and male (♂, right) mice.
Each panel displays three experimental groups: Control, FA+CBP Milk, and Positive Control. Female and male mice are presented separately to highlight sex-specific responses. Individual data points are overlaid on bar graphs to illustrate variability within each sex. Statistical significance versus the Control group is indicated by asterisks (*$p < 0.05$; ** $p < 0.01$ vs. Control, ns: not significant.).
